# Case Report: Olfactory loss as the sentinel manifestation of type 2 chronic rhinosinusitis without polyps: response to IL-4Rα inhibition with stapokibart - a case series

**DOI:** 10.3389/fimmu.2026.1936613

**Published:** 2026-09-30

**Authors:** Hua Cai, Tao Zhou, Juan-Juan Hu, Sha-Zhou Li, Xiang-Ning Cheng, Yu-Zhe Hao, Jian-Jun Chen, Yao Hu, Liu-Qing Zhou

**Affiliations:** 1 Department of Otorhinolaryngology, Union Hospital, Tongji Medical College, Huazhong University of Science and Technology, Hubei, Wuhan, China; 2 Hubei Province Clinical Research Center for Deafness and Vertigo, Wuhan, China; 3 Department of Otorhinolaryngology, The Central Hospital of Wuhan, Huazhong University of Science and Technology, Wuhan, China

**Keywords:** allergic rhinitis, case series, chronic rhinosinusitis without nasal polyps, olfactory dysfunction, stapokibart

## Abstract

Chronic rhinosinusitis without nasal polyps (CRSsNP) is traditionally associated with type 1 inflammation, but emerging evidence reveals a subset of patients with a type 2 endotype driven by IL-4 and IL-13, often presenting with prominent olfactory dysfunction and responding poorly to conventional therapy. We report three cases of type 2 CRSsNP with comorbid allergic rhinitis (aged 29–30 years, two males, one female) who presented with debilitating olfactory loss as the sentinel symptom. All had elevated type 2 biomarkers, absence of nasal polyps, and CT evidence of sinusitis. After failure of intranasal corticosteroids, they received stapokibart (600 mg loading dose, then 300 mg every two weeks for 16 weeks). Olfactory improvement occurred within two weeks and was sustained through week 16; SNOT-22 scores decreased by approximately 90%, and Lund-Mackay scores decreased to 0 (Cases 1 and 2) and 1 (Case 3). Serum total IgE decreased in all patients; eosinophil counts decreased in two but paradoxically increased in one. No serious adverse events occurred. Type 2 CRSsNP with prominent olfactory dysfunction is a distinct, treatable phenotype. Stapokibart rapidly improves olfactory function in patients refractory to conventional therapy, supporting an endotype-driven biologic strategy for CRS.

## Introduction

Chronic rhinosinusitis (CRS) constitutes a persistent inflammatory process affecting the mucosa of the nasal cavity and paranasal sinuses. Its clinical presentation includes nasal congestion, discharge, reduced olfactory function, and facial pressure. CRS is traditionally phenotypically classified into CRS with nasal polyps (CRSwNP) and CRS without nasal polyps (CRSsNP) based on endoscopic findings. However, this phenotypic classification does not fully capture the underlying inflammatory endotypes. CRSsNP accounts for approximately 75% to 90% of all CRS cases, yet it has not been the primary focus of endotype research, partly due to its heterogeneous presentation and the absence of a consensus tissue biopsy site for standardized assessment (1). All three patients in this report also carried a diagnosis of allergic rhinitis, a common comorbidity of type 2 CRS. While CRSwNP is predominantly driven by type 2 inflammation characterized by eosinophilic infiltration and elevated IL-4, IL-5, IL-13, and IgE (2), CRSsNP was historically associated with a type 1 inflammatory profile. However, this binary view has been increasingly challenged as evidence reveals considerable endotypic heterogeneity (3). Recent studies have demonstrated significant variability in inflammatory biomarkers when comparing inferior turbinate, uncinate process, and ethmoid sinus tissue obtained from the same patients with CRSsNP, with higher concentrations of type 2 and type 3 biomarkers detected in the less accessible ethmoid sinuses (4). These findings suggest that a subset of CRSsNP patients harbors occult type 2 inflammation that may remain clinically unrecognized yet contribute to refractory symptoms, particularly olfactory dysfunction.

Despite adherence to guideline-directed medical therapy, optimal management of type 2 CRSsNP with predominant olfactory symptoms remains undefined, and such patients often exhibit incomplete responses to conventional treatment. Herein, we report three cases of type 2 CRSsNP with comorbid allergic rhinitis presenting with debilitating olfactory loss that persisted despite intranasal corticosteroids therapy, who achieved rapid and sustained olfactory recovery with stapokibart (CM310), a novel anti-IL-4Rα monoclonal antibody. Stapokibart has been approved in China for the treatment of chronic rhinosinusitis with nasal polyps and seasonal allergic rhinitis (5, 6).

## Case presentation

### Case 1

A 30-year-old female presented with a six-month history of progressive olfactory loss as the predominant symptom, accompanied by nasal congestion and rhinorrhea. She had been treated with intranasal corticosteroids combined with oral antihistamines, but her sinonasal symptoms remained poorly controlled and olfactory dysfunction persisted. She had no history of asthma, aspirin sensitivity, or prior sinus surgery. Anterior rhinoscopy revealed turbinate hypertrophy with pale and edematous nasal mucosa (**Figure 1A**). Allergy testing confirmed sensitization to house dust mites, and the patient was diagnosed with comorbid allergic rhinitis. Nasal endoscopy revealed pale, edematous mucosa with moderate swelling in the bilateral olfactory clefts, without evidence of polypoid lesions. The middle meatus was patent bilaterally. Computed tomography (CT) of the paranasal sinuses demonstrated patchy soft tissue opacities predominantly involving the central compartment, including the bilateral maxillary, ethmoid, and sphenoid sinuses (**Figure 1B**), with a Lund-Mackay score of 6. Laboratory evaluation revealed an elevated serum total IgE of 70.10 KU/L (reference range: < 60 KU/L) and peripheral eosinophilia of 0.73 × 10^9^/L (reference range: 0.02–0.52 × 10^9^/L), with an eosinophil percentage of 10.4% (reference range: 0.4%–8%). Baseline symptom scores included a SNOT-22 score of 48, a Visual Analogue Scale (VAS) for smell loss of 6, a nasal congestion score (NCS) of 3, and an olfactory identification score (SSIT, 16-point scale) of 8. No family history of atopy, asthma, or autoimmune diseases was reported. The patient denied significant psychosocial stressors.

**Figure 1:**
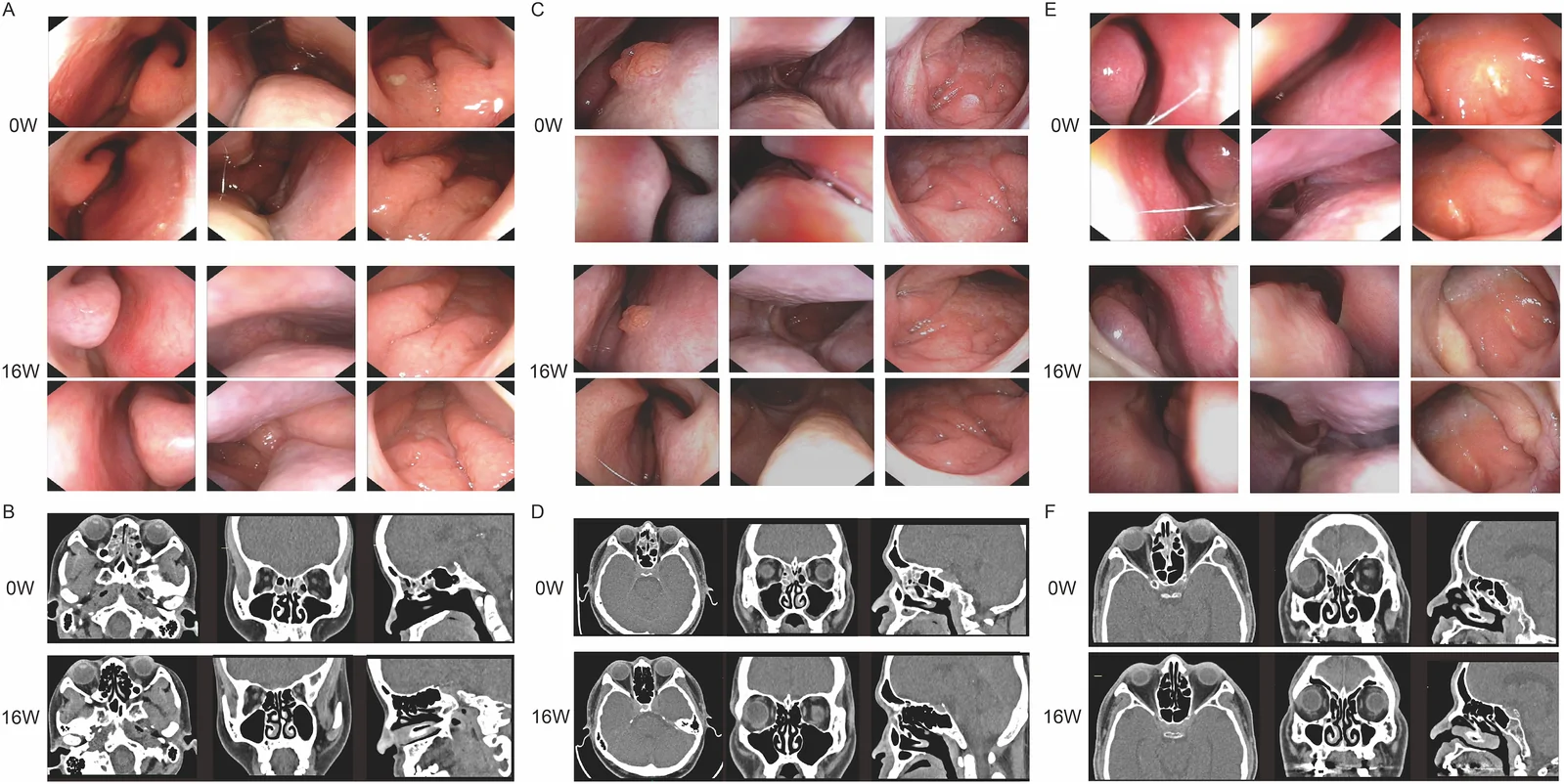
Radiological and endoscopic findings at baseline and week 16. CT scans **(B, D, F)** and endoscopic views of the olfactory cleft/middle meatus **(A, C, E)** at baseline (0W) and after 16 weeks of stapokibart treatment for the three patients [Case 1: Panels **(A, B)**; Case 2: Panels **(C, D)**; Case 3: Panels **(E, F)**]. **(A, C, E)** Endoscopy at baseline revealed mucosal swelling and/or discharge in the olfactory cleft, which showed marked resolution by week 16. **(B, D, F)** CT imaging at baseline showed patchy soft tissue opacities involving the maxillary and ethmoid sinuses, which resolved completely by week 16.

### Case 2

A 30-year-old male presented with a one-year history of isolated olfactory loss as the initial and most bothersome symptom, associated with recurrent nasal itching and paroxysmal sneezing. Despite treatment with intranasal corticosteroids (mometasone furoate) combined with oral antihistamines, his sinonasal symptoms remained poorly controlled, and olfactory dysfunction persisted. He had no history of asthma or nasal polyps. Allergy testing confirmed sensitization to house dust mites and molds (specific IgE class 3), and the patient was diagnosed with comorbid allergic rhinitis.

Nasal endoscopy revealed mild to moderate mucosal swelling in the olfactory cleft with mucoid discharge, without polypoid lesions (**Figure 1C**). CT imaging demonstrated patchy soft tissue opacities involving the left frontal sinus and bilateral ethmoid and maxillary sinuses, with central predominance (**Figure 1D**), and a Lund-Mackay score of 8. Laboratory evaluation revealed an elevated serum total IgE of 79.50 KU/L and peripheral eosinophil count of 1.5 × 10^9^/L (eosinophil percentage: 2.4%). Baseline symptom scores included a SNOT-22 score of 38, a VAS for smell loss of 10, an NCS of 1, and an olfactory identification score (SSIT) of 1, indicating severe olfactory dysfunction. There was no family history of allergic diseases, and the patient reported no psychosocial concerns.

### Case 3

A 29-year-old male presented with several years of nasal obstruction and a three-month history of progressive olfactory loss, which was his most bothersome symptom. He had been treated with intranasal corticosteroids intermittently, but his sinonasal symptoms remained poorly controlled and olfactory dysfunction persisted. He had no history of asthma or prior sinus surgery. Anterior rhinoscopy revealed turbinate hypertrophy with pale and edematous nasal mucosa. Allergy testing confirmed sensitization to house dust mites, and the patient was diagnosed with comorbid allergic rhinitis. Nasal endoscopy revealed diffuse mucosal swelling in the olfactory cleft and middle meatus, without polypoid lesions (**Figure 1E**). CT imaging demonstrated patchy soft tissue opacities involving the bilateral maxillary and ethmoid sinuses (**Figure 1F**), with a Lund-Mackay score of 7. Laboratory evaluation revealed a serum total IgE of 29.60 KU/L and peripheral eosinophil count of 0.54 × 10^9^/L (eosinophil percentage: 9.4%). Baseline symptom scores included a SNOT-22 score of 57, a VAS for smell loss of 3, an NCS of 3, and an olfactory identification score (SSIT) of 5. Family history was unremarkable for atopic conditions. The patient denied any major life stressors.

### Diagnostic considerations

The main diagnostic challenge in these cases was distinguishing type 2 CRSsNP from other causes of olfactory dysfunction, including post-viral olfactory loss and neurodegenerative disorders. The presence of chronic sinonasal symptoms, characteristic CT findings of sinus opacification, and elevated type 2 biomarkers (total IgE and/or peripheral eosinophilia) supported the diagnosis of inflammatory sinonasal disease rather than neurological causes. Other diagnoses considered included chronic bacterial sinusitis (excluded by lack of purulence and negative cultures) and allergic fungal rhinosinusitis (excluded by absence of fungal elements on endoscopy and characteristic serpiginous hyperattenuation on CT). The absence of nasal polyps on endoscopy further distinguished these cases from CRSwNP, while the elevated type 2 biomarkers and comorbid allergic rhinitis pointed toward a type 2 endotype of CRSsNP.

## Materials and methods

### Patients

Three patients with refractory olfactory loss, elevated type 2 biomarkers (serum IgE and/or eosinophilia), no nasal polyps on endoscopy, and CT evidence of chronic sinusitis were enrolled. All patients provided written informed consent.

### Treatment protocol and outcome measures

After failure of intranasal corticosteroids, all patients received a 600 mg subcutaneous loading dose of stapokibart (CM310), followed by 300 mg every two weeks for 16 weeks, in combination with ongoing intranasal corticosteroids.

Clinical outcomes were assessed at baseline and at weeks 2, 4, 6, 8, 12 and 16 using the following instruments:

- 22-item Sinonasal Outcome Test (SNOT-22)
- Visual Analogue Scale (VAS) for smell loss (0–10)
- Nasal Congestion Score (NCS) (0–3)
- Olfactory identification score using the SSIT (16-point scale, with higher scores indicating better function)
- Lund-Mackay CT score: Radiological assessment of sinus opacification on paranasal sinus CT was performed using the Lund-Mackay staging system (7). Each of the six paired anatomical sites was graded bilaterally: the maxillary, anterior ethmoid, posterior ethmoid, sphenoid, and frontal sinuses, and the ostiomeatal complex (OMC). Each sinus was scored as 0 (no opacification), 1 (partial opacification), or 2 (total opacification), whereas the OMC was scored dichotomously as 0 (not occluded) or 2 (occluded). The total Lund-Mackay score therefore ranged from 0 to 24 per patient (12 points per side), with higher values indicating more extensive sinus opacification. All baseline and week-16 CT images were re-evaluated in a paired, randomized order by a board-certified head-and-neck radiologist blinded to the clinical visit, treatment assignment, and patient identity; any discrepancy was adjudicated by consensus with a senior otorhinolaryngologist.
- Laboratory markers: serum total IgE and peripheral eosinophil counts

### Ethical approval

Ethical approval was obtained from the Ethics Committee of Union Hospital, Tongji Medical College, Huazhong University of Science and Technology (UHCT-IEC-SOP-016-03-01). All patients provided written informed consent for publication.

## Results

Following initiation of stapokibart therapy, all three patients demonstrated rapid and sustained clinical improvement. Notably, olfactory recovery occurred within the first two weeks of treatment, with near-complete restoration of smell function by week 4, as reflected by the SSIT scores increasing from severely impaired baselines to near-normal or normal levels. Patient-reported outcomes, including SNOT-22, VAS, and NCS, showed consistent downward trends paralleling the olfactory gains.

Case 1 achieved an olfactory score of 16 by week 4 (from 8 at baseline), with SNOT-22 improving from 48 to 5 and VAS from 6 to 0 by week 16. Her peripheral eosinophil count paradoxically increased from 0.73 × 10^9^/L to 0.92 × 10^9^/L despite marked symptom resolution, consistent with the known paradoxical eosinophilia associated with IL-4Rα blockade.

Case 2 with the most severe baseline olfactory impairment (SSIT score of 1, VAS of 10), showed a more gradual but equally impressive recovery, reaching an SSIT score of 15 by week 16 (SNOT-22: 38→4; VAS: 10→0).

Case 3 demonstrated the most rapid response, achieving an SSIT score of 16 by week 8 and a SNOT-22 reduction from 57 to 6 by week 16.

The longitudinal changes in SNOT-22 (A), olfactory VAS (B), nasal congestion score (C), olfactory identification on the SSIT (D), Lund-Mackay CT score (E), peripheral eosinophil count (F), eosinophil percentage (G), and serum total IgE (H) for all three patients are shown in **Figure 2**.

**Figure 2:**
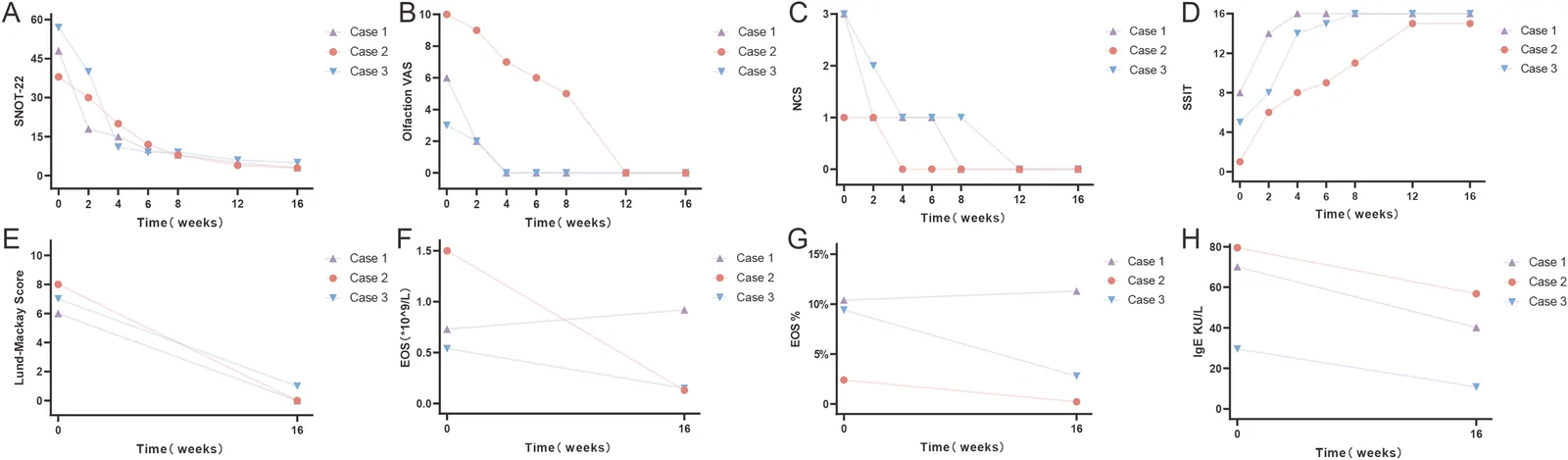
Longitudinal changes in clinical outcome measures during stapokibart treatment. **(A)** SNOT-22 scores. **(B)** Visual Analogue Scale (VAS) for smell loss (0–10, with 10 indicating the most severe impairment). **(C)** Nasal Congestion Score (NCS, 0–3). **(D)** SSIT olfactory identification score (16-point scale). **(E)** Lund-Mackay CT score (0–24, assessed at baseline and week 16 only). **(F)** Peripheral eosinophil count (×10^9^/L). **(G)** Peripheral eosinophil percentage (%). **(H)** Serum total IgE (KU/L). All three patients showed rapid and sustained improvement across all measures, with olfactory recovery (SSIT) beginning within the first 2 weeks. SNOT-22, 22-item Sinonasal Outcome Test; VAS, Visual Analogue Scale; NCS, Nasal Congestion Score; SSIT, Sino-Nasal Symptom Interview Test olfactory identification subscale; W, week.

Radiologically, all three patients showed substantial improvement on follow-up CT imaging at week 16 (**Figures 1B, D, F**): Lund-Mackay scores decreased from 6, 8, and 7 at baseline to 0.

(Cases 1 and 2) and 1 (Case 3), with complete radiological resolution in two patients and only minimal residual mucosal thickening (score of 1 in a single sinus) in the third. A *post-hoc* conservative re-interpretation of the CT images, scoring any residual mucosal thickening as 1 rather than 0, still yielded a reduction of ≥5 points in all cases, exceeding the established MCID of 4.95 (8). Nasal endoscopy at week 16 confirmed marked resolution of mucosal swelling in all patients (**Figures 1A, C, E**), with no evidence of polyp formation throughout the treatment course.

Laboratory markers demonstrated variable but overall favorable trends. Serum total IgE decreased in all three patients: Case 1 from 70.10 KU/L to 40.2 KU/L, Case 2 from 79.50 KU/L to 56.80 KU/L, and Case 3 from 29.60 KU/L to 10.80 KU/L. Peripheral eosinophil counts decreased in Case 2 (1.5 → 0.13 × 10^9^/L) and Case 3 (0.54 → 0.15 × 10^9^/L), but increased in Case 1 (0.73 → 0.92 × 10^9^/L), consistent with the paradoxical eosinophilia observed in some patients receiving anti-IL-4Rα therapy.

All three patients reported high satisfaction with treatment outcomes, particularly the restoration of olfactory function and associated improvement in quality of life. No adverse events were observed during the 16-week treatment period in any patient.

All three patients were interviewed regarding their treatment experience. They uniformly reported that olfactory loss had been the most distressing symptom, affecting their appetite, safety awareness, and overall quality of life. Prior to stapokibart therapy, they had tried intranasal corticosteroids with limited benefit and expressed frustration with persistent smell dysfunction. After initiating stapokibart, they noticed improvement in smell function within the first two weeks, which they described as “life-changing.” They reported high satisfaction with the subcutaneous injection regimen and expressed willingness to continue treatment if needed. No patient reported discomfort or injection-site reactions during the 16-week treatment period.

Treatment adherence was confirmed by outpatient injection records, with all patients receiving all scheduled doses without interruption. No patient reported missing any dose throughout the 16-week regimen.

## Discussion

The management of CRS remains clinically challenging, particularly for patients with type 2 inflammation who fail to respond adequately to conventional therapies. First-line treatment for CRS typically includes intranasal corticosteroids and saline irrigation, with short courses of systemic corticosteroids reserved for severe exacerbations. Endoscopic sinus surgery is indicated for patients refractory to medical management, but long-term outcomes are often unsatisfactory, with high rates of recurrence, persistent olfactory dysfunction, and the need for revision surgery. While these challenges are well-documented in CRS with nasal polyps (CRSwNP) (6), patients with CRS without nasal polyps (CRSsNP) and underlying type 2 inflammation face similar therapeutic difficulties—yet remain underrecognized due to the historical association of non-polypoid disease with type 1 inflammation (3, 9).

Biologics targeting type 2 inflammation have revolutionized the treatment landscape for CRSwNP and AR, but their role in CRSsNP has remained undefined due to the historical association of non-polypoid disease with type 1 inflammation (2, 10). Type 2 inflammation, driven by IL-4, IL-5, and IL-13, plays a central role in the pathogenesis of both CRSwNP and a subset of CRSsNP, particularly those presenting with prominent olfactory dysfunction and elevated type 2 biomarkers (11, 12). Stapokibart (CM310) is a novel humanized monoclonal antibody that binds the interleukin-4 receptor alpha subunit (IL-4Rα), thereby blocking both IL-4 and IL-13 signaling pathways—key drivers of type 2 inflammation and eosinophilic infiltration (13). Mechanistically, stapokibart interacts with a distinct conformational epitope on IL-4Rα located closer to IL-4’s natural binding site (residues M39A, S95A, L135A), distinguishing it from other anti-IL-4Rα biologics (14, 15). *In vitro* studies have further demonstrated that stapokibart effectively prevents IL-4 and IL-13/IL-13Rα1 binding to IL-4Rα, with neutralizing activity comparable to or exceeding that of other biologics targeting the same pathway (15). It was approved in China for the treatment of CRSwNP in December 2024 and for seasonal allergic rhinitis in February 2025 (10). Clinical studies have shown that stapokibart significantly improves nasal and ocular symptoms in type 2 inflammatory conditions, with a favorable safety profile in severe eosinophilic CRSwNP and seasonal allergic rhinitis (13, 16). However, its role in non-polypoid CRS has remained largely unexplored.

In the present cases, the three patients with type 2 CRSsNP presenting with debilitating olfactory loss as the sentinel symptom achieved rapid and sustained olfactory recovery following stapokibart therapy. Notably, olfactory improvement was observed within two weeks, with near-complete resolution by week four—a timeframe considerably shorter than the 24-week timelines typically reported for olfactory improvement with established biologics in CRSwNP (9, 17). This rapid recovery may be attributed to the potent and early reduction of mucosal edema and inflammation in the olfactory cleft facilitated by dual IL-4/IL-13 pathway inhibition. In contrast to CRSwNP, where olfactory dysfunction often results from mechanical obstruction by polypoid tissue, olfactory impairment in type 2 CRSsNP is likely driven predominantly by inflammatory infiltration of the olfactory epithelium in the absence of macroscopic polyps. This inflammatory pathology may be more rapidly reversible upon effective suppression of type 2 cytokines, potentially explaining the accelerated olfactory recovery observed in our patients. The co-diagnosis of allergic rhinitis in all three patients further supports the type 2 inflammatory nature of their disease, as allergic rhinitis shares common pathophysiological pathways with type 2 CRS.

Several limitations of the present study should be acknowledged. First, the sample size is small (n=3), which precludes formal statistical inference and limits the generalizability of our findings. Second, the follow-up period of 16 weeks is relatively short, and we therefore cannot yet assess the durability of response, the potential for relapse after discontinuation, or long-term safety beyond this window. The optimal duration of stapokibart therapy for type 2 CRSsNP remains unknown, and questions regarding long-term durability of response, potential for relapse upon discontinuation, and ideal maintenance strategies require further investigation. Additionally, while all three patients demonstrated elevated type 2 biomarkers, the diagnosis of type 2 CRSsNP was inferred from peripheral markers rather than confirmed by tissue eosinophilia or local cytokine measurement, highlighting the ongoing challenge of endotyping non-polypoid disease in the absence of consensus tissue for biopsy (1, 4).

Notably, although all three patients achieved marked clinical and radiological improvement, divergent peripheral eosinophil responses were observed: Case 1 showed a paradoxical increase from 0.73 × 10^9^/L at baseline to 0.92 × 10^9^/L at week 16 (eosinophil percentage: 10.4% to 11.3%), whereas Cases 2 and 3 demonstrated decreases from 1.5 × 10^9^/L to 0.13 × 10^9^/L and from 0.54 × 10^9^/L to 0.15 × 10^9^/L, respectively. This phenomenon, known as paradoxical eosinophilia, has been increasingly recognized during anti-IL-4Rα therapy and is thought to result from reduced eosinophil apoptosis due to IL-5 signaling that becomes relatively unopposed following IL-4/IL-13 blockade. Importantly, this transient laboratory finding is not necessarily indicative of treatment failure, as it may occur alongside significant symptom improvement, as demonstrated in our cases. The divergent responses among the three cases—an increase in Case 1 (with baseline eosinophilia) versus decreases in Cases 2 and 3 (with normal or mildly elevated baseline eosinophils)—suggest that baseline eosinophil status may influence the direction of peripheral eosinophil changes during IL-4Rα blockade, underscoring the importance of individualized monitoring. Nevertheless, this observation highlights the need for continuous monitoring of peripheral eosinophil counts throughout the treatment course, not only to detect potential hypereosinophilia-related adverse events but also to better characterize the individual patient’s evolving inflammatory profile and guide subsequent management decisions.

## Conclusion

In conclusion, these cases demonstrate that type 2 CRSsNP presenting with predominant olfactory loss represents a distinct and treatable phenotype. Stapokibart, through dual blockade of IL-4 and IL-13 signaling via IL-4Rα inhibition, offers a potent therapeutic option for this underrecognized patient population. The rapid olfactory recovery observed—within weeks rather than months—suggests that early intervention with targeted biologic therapy may prevent chronic olfactory dysfunction and improve quality of life. Larger controlled studies with extended follow-up are warranted to further define its efficacy, optimal dosing, and long-term safety profile in non-polypoid CRS, and to establish criteria for endotype-driven patient selection that transcend traditional phenotype-based paradigms.

## Data Availability

The original contributions presented in the study are included in the article/supplementary material. Further inquiries can be directed to the corresponding authors.
